# Effect of Vibration Procedure on Particle Distribution of Cement Paste

**DOI:** 10.3390/ma16072600

**Published:** 2023-03-24

**Authors:** Jia Ke, Zhonghe Shui, Xu Gao, Xibo Qi, Zihang Zheng, Shaolin Zhang

**Affiliations:** 1 International School of Materials Science and Engineering, Wuhan University of Technology, Wuhan 430070, China; 2 State Key Laboratory of Silicate Materials for Architectures, Wuhan University of Technology, Wuhan 430070, China; 3 Wuhan University of Technology Advanced Engineering Technology Research Institute of Zhongshan, Xiangxing Road 6, Zhongshan 528400, China; 4 School of Materials Science and Engineering, Wuhan University of Technology, Wuhan 430070, China; 5 School of Civil Engineering and Architecture, Wuhan University of Technology, Wuhan 430070, China; 6 China Construction Third Bureau First Engineering Co., Wuhan 430070, China

**Keywords:** vibration procedure, particle distribution, tagged materials, cement paste

## Abstract

Vibration procedures significantly affect the performances of cement-based materials. However, studies on the distribution of certain particles within cement-based materials are limited due to the complexity and difficulty of identifying each specific particle. This paper presents a new method for simulating and quantifying the movements of particles within cement paste through the use of “tagged materials”. By separating the tagged particles from the cement paste after vibration, the distribution of the particles in the cement paste can be calculated statistically. The effect of the vibration time and frequency, fresh behavior, and powder characteristics of cement paste on particle motions are investigated. The results demonstrate that when the vibration exceeds 1800 s, it induces a significant uneven dispersion of microparticles. This effect is more pronounced at low viscosities (<1 Pa·s) of cement paste or high vibration frequencies (>200 Hz). Larger and denser particles exhibit greater dispersion. This method provides a valuable tool for investigating the theory of particle motion in cement paste, which is crucial for understanding the influence of vibration on the properties of cement-based materials.

## 1. Introduction

Cement-based materials are most widely used as concrete structures, dominating in buildings and infrastructure [1]. During casting, vibration is frequently used to maintain the ideal dispersion and density of cement-based materials, as vibration eliminates air bubbles and rearranges the particle composition in concrete [2]. Construction guides and specifications, such as the ACI Material Journal, have shown how vibration should be performed to achieve dense concrete [3]. However, the vibration process is usually designed based on previous experiences. These vibration procedures are different for different conditions and projects. Moreover, it should be noted that inappropriate vibration can cause a reduction in the strength and durability of cement-based materials [4].

The impact of mechanical vibration on the performance of cement-based materials has been extensively studied, as it is the most commonly encountered type of vibration during construction and directly affects the quality and service life of concrete structures [5,6]. Vibration is a macroscopic physical process, and the microstructure of cement-based materials is altered during this process [7]. Understanding how the structure and hydration mechanisms of cement-based materials change during vibration is crucial for comprehending the changes in their properties [8,9].

It has been concluded that in cement-based materials, vibration accelerates the hydration and dissolution of cementitious particles, leading to the rapid formation of the cement matrix. This process enhances the strength of cement-based materials but may also result in the formation of microcracks. More specifically, vibration can readily influence microstructure development and increase the possibility of microdefects [2,7,10]. Another widely accepted viewpoint is that the yield stress of cement-based materials significantly decreases upon vibration as a result of the breaking of the interlocking of coarse aggregates due to the movement of cement-based materials in response to vibration, which disrupts the attraction between the cement and other microscopic particles and reduces the internal friction of the cement paste [4]. The improvement of mechanical properties by vibration is often attributed to the rearrangement of particles within cement-based materials. It is widely accepted that the composition and structure of a material dictate its properties [11,12]. Particularly for the particle distribution characteristics of cement-based materials under vibration, particle rearrangement greatly determines the performance of cement-based materials [13]. The improvement in the compactness of cement-based materials can be confirmed by detecting the porosity of cement-based materials after vibration treatment, typically tested using mercury intrusion porosimetry (MIP) [14].

Currently, it is still a challenging issue to visually observe the changes of particle distribution in cement-based materials after vibration. Many scholars have studied the movement characteristics of the aggregates and fiber in cement-based materials during vibration. In steel-fiber-reinforced concrete (SFRC), researchers usually evaluate the fiber distribution by statistically analyzing the area, orientation, or count of fibers in different cross-sectional regions [15,16]. By washing out different parts of the coarse aggregate of concrete after vibration for weighing and statistical calculations, Mohammad derived the distribution characteristics of coarse aggregate at different vibration intensities and viscosities of concrete and proposed a vibration strategy to control fresh high-fluidity concrete to maintain homogeneity and workability [17,18]. Petrou used radioactive metal spheres as tagged aggregates and used a scintillation camera to track their position in a concrete mix under vibration, simulating the movement of aggregates in concrete-based materials under vibration in real time [4].

Compared to coarse aggregates and fibers, cement particles are much smaller, and it is more challenging to characterize their distribution. Due to limitations in testing methods and detection means, the distribution changes of small-sized particles such as cement particles and mineral admixtures in cement-based materials after vibration are often overlooked. By examining the strength, porosity, and elemental distribution of the various layers of cement test blocks, Ling was able to detect and characterize the inhomogeneous distribution of various microparticles and confirmed that this inhomogeneity is related to the mechanical properties of hardened cement paste [2]. This means that the distribution of microparticles is important to the properties of cement-based materials. At the current stage, numerical computer simulations are often unable to fully simulate actual operating conditions or experimental conditions, and the use of isotopic labeling is costly. There is a need for a simple and quantifiable method to characterize the motion of particles in cement-based materials under vibration.

In this paper, “tagged” materials are used to simulate cement particles and other particles in the paste [4]. These materials are insoluble in water and have a similar particle size as cement particles. After vibration, the tagged materials are separated from different parts of the cement block by washing with water and screening, allowing us to observe the particle distribution in the cement paste. The feasibility of using tagged materials to explore the distribution and movement patterns of cement particles under vibration is evaluated, and the correlation between the vibration procedure (time and frequency) and the properties of the cement paste are discussed, which is essential for optimizing the design and performance of cement-based materials.

## 2. Materials and Methods

Portland cement type I 42.5 according to Chinese National Standard GB 175-2007 was used as the binder. The water consumption of the cement was 26.1% [19]. The following were used to produce the paste samples: Fe powder with a particle size between 25 and 100 μm and a density of about 7.85 gr/cm³; C powder with a particle size distribution of 25–100 μm and a density of about 1.60 gr/cm³. The following were used as tag materials: silicon carbide powder with a particle size of 25–100 μm and a density of about 3.23 gr/cm³; boron carbide (B_4_C, a ceramic material composed of boron and carbon atoms that has good chemical resistance and does not interact with water or cement) powder with a particle size of 25–100 μm and a density of about 2.52 gr/cm³. A polycarboxylic ether-based superplasticizer (SP) was used to adjust the workability. The SP had a solid content of about 20% and a water-reducing rate of more than 30% [20,21].

**Figure 1:**
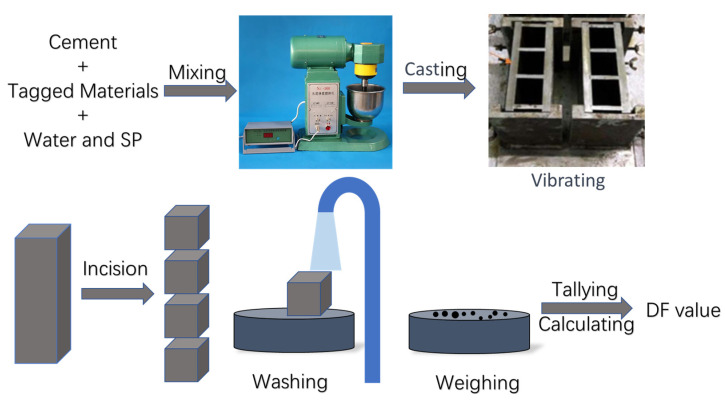
Schematic diagram of main experimental procedures.

A method aimed at quantifying the trend and distribution of particles in cement-based materials under vibration is proposed. The experimental procedure is depicted in Figure 1. Solid particles of a similar size to cement particles were selected to simulate the motion of cement particles in the cement paste. These particles exhibit excellent chemical resistance, and they are not soluble in water. Therefore, they can be separated from different parts of the cement paste by sieving through a mesh with a smaller pore size than the particles. A four-degrees-of-freedom vibration table was used to provide different vibration conditions, with an iron vertical triple mold fixed on the vibration table. The mold size was 40 × 40 × 160 mm (height), with a thickness of 10 mm for the long side and 7 mm for the short side. After the tagged particles were screened through a 25 μm sieve, they were mixed into the cement paste according to the designed proportions and poured into the mold [22]. The vibration process was completed by adjusting the vibration table. Before the cement paste hardened, the mold was removed from the shaking table, and the long-side plate was taken off. The cement paste was then divided into four parts using 0.1 mm thick metal sheets, to allow for subsequent testing of different parts of the paste. The mass of each part was recorded as “m”. Each part was rinsed with running water, and the cement paste was sieved through a 25 μm sieve to collect the particles that remained on the sieve. After drying under vacuum, the mass of these particles was weighed and recorded as “d” for further analysis. The ratio of d to m was defined as DR, and $\bar{\mathrm{DR}}$ was defined as the ratio of the total mass of the dopant material to the total mass of the cement paste (as illustrated in Equations (1) and (2)) [17,23]:

(1) $$\mathrm{DR}=d/m=\frac{\mathrm{weight}\;\mathrm{of}\;\mathrm{dopant}\;\mathrm{materials}\;\mathrm{of}\;\mathrm{the}\;\mathrm{layer}}{\mathrm{weight}\;\mathrm{of}\;\mathrm{total}\;\mathrm{mass}\;\mathrm{of}\;\mathrm{the}\;\mathrm{layer}}$$

(2) $$\bar{\mathrm{DR}}=d_{\mathrm{total}}/{m\;}_{\mathrm{total}}=\frac{\mathrm{total}\;\mathrm{weight}\;\mathrm{of}\;\mathrm{dopant}\;\mathrm{materials}\;}{\mathrm{total}\;\mathrm{weight}\;\mathrm{of}\;\mathrm{the}\;\mathrm{cement}\;\mathrm{paste}}$$

To quantitatively characterize the movement and migration of tagged materials in cement-based materials under different conditions, a calculation method has been defined. This method assigns discrete significance to the specific gravity of the DR for different layers and defines it as DF. The DF is used to measure the degree of dispersion of the tagged materials in each group (as illustrated in Equation (3)) [15,17]:

(3) $$DF=\sqrt{\frac{\sum_{i=1}^{i=n}{\left(1-x_{i}\right)}^{2}}{n}}\;(x_{i}=\frac{DR_{i}}{\left(\bar{DR}\right)})$$

The mix proportions used in this study for each section are listed in Table 1.

## 3. Results and Discussion

### 3.1. Validation of the Proposed Method

**Figure 2:**
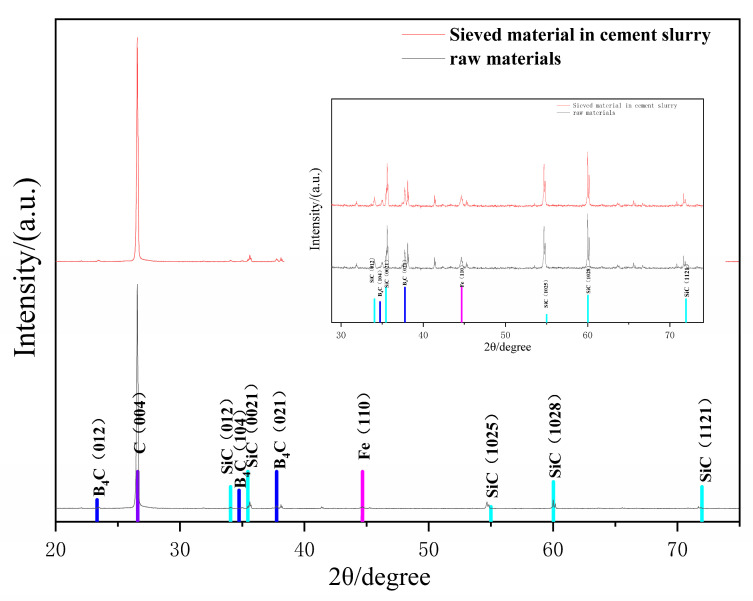
XRD results of the tagged material mixture with the sieve residue.

For the method used to quantitatively characterize the trend of particle distribution in cement-based materials under vibration, the following experiment is designed to illustrate its feasibility and reliability. All kinds of tagged particles were mixed and sifted through a screen with a 25-micron pore size. The mixture was weighed and subsequently added to the cement paste, which was then thoroughly mixed. After that, the cement paste, as well as the mixing container, was slowly rinsed through the same screen with running water. The mass of the particles collected through the sieve residue is weighed and compared to the mixture of tagged particles. The composition of the mixture is given in Table 1. Figure 2 shows the XRD results of the tagged material mixture with the sieve residue.

As a result, the mass of the tagged particles recovered was 93.2% of the original mass, by means of sieving, as water and cement particles reacted and combined to form a suspension. They adhered to each other. It could be flushed from the screen point with the flowing water, and other insoluble materials were left on the screen, as the materials used were not chemically or physically reactive with water, most of the tagged particles can be collected. The XRD plots of the original sample and the sample rinsed and sieved from the paste showed that the rinsed sample had basically the same XRD spectrum as the original sample. The (004) crystalline surface of the toner had a meritocratic orientation. Strong diffraction peaks appeared and showed high absorption values in XRD, and the peak intensity of the rest of the material is basically the same, indicating that the basic composition of the material obtained after the test process was the same as the raw material. The tagged materials selected for this experiment were easy to separate from the cement paste and maintain their original physical properties. It means that the tagged particles collected from different parts of the cement slurry can be considered representative of all tagged particles in certain parts. The feasibility of using flushing sieve residuals to quantify how much tagged material is available for different portions of cement-based materials was verified.

### 3.2. Effect of Vibration Duration

The relationship between vibration time and the distribution of the tagged particles is investigated. The materials ratios used in this phase of the experiment are shown in Table 1. The selected fundamental frequency of 200 Hz and the gravitational acceleration of 5G (average displacement—0.06 mm) and vibration times are selected as 0 s, 30 s, 90 s, 180 s, 360 s, 1080 s, 2160 s, 3600 s, and 5400 s.

**Figure 3:**
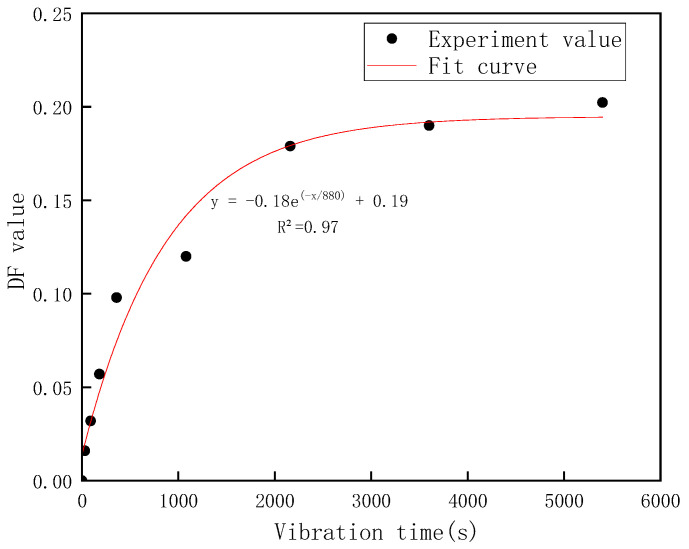
Variation of DF value with vibration time.

Figure 3 shows the graph of DF values changing with vibration time, it can be seen that with the increase in vibration duration, the overall DF calculation value gradually increases. The DF value is used to characterize the dispersion of the distribution of tagged particles between the different layers. At this time, because of the occurrence of vibration, the yield stress of the cement paste decreased, and the particles were subjected to gravity to settle in the paste, resulting in a certain uneven distribution within the paste, and the result shows that the DF increases with time. When the vibration time was 0, the calculated DF value was very close to 0, which was in accordance with the expected result. When the cement paste was not subject to vibration, it possessed a certain yield stress. According to the research of Petrous et al. [4], even in the absence of vibration, it was difficult for high-quality aggregate or materials with higher densities than the aggregate to induce motion, even when their volume was the same. The added material was uniformly distributed in the paste at the vibration time of 0. As time went on, the rate at which the discrete distribution of marked particles increased gradually slowed down as the settle range of particles was limited, and the internal structure of the cement paste changed, causing a gradual change in yield stress and a gradual slowdown in the trend of the movement of material particles. These factors all contributed to the gradual decline in the growth rate of the DF value. The use of tagged particles has allowed researchers to discover that small particles with similar size and density to cement particles can move within cement paste when subjected to vibration. Even short periods of vibration, less than 180 s, can cause these particles to move vertically in a directional manner, although this may not be easily observable. However, after prolonged vibration for 2000 s or longer, this directional motion becomes more apparent, resulting in phenomena such as bleeding and bubble discharge in the cement substrate [9,24]. This suggests that long-term vertical vibration can cause the distribution of substances other than solid particles to become oriented.

In this experiment, the movement of particles similar in size to the cement and mineral admixture particles in the cement paste by using tagged particles is studied. It was found that the particles within the cement slurry underwent continuous directional migration upon exposure to vibration; however, the trend of dispersion of particle distribution due to this migration gradually slowed down over time. The result demonstrates the feasibility of this method for simulating and quantifying the motion of particles similar in size to cement and provides a solid experimental foundation for further experiments

### 3.3. Influence of Paste Flowability and Viscosity

Based on the above experiments, the method shown in Figure 1 is used to evaluate the relationship between the fluidity of cement paste and the viscosity and particle distribution in the vibrating state. The distribution ratio of the tagged particles in different parts of the cement paste block is obtained. Different viscosities and fluidities were applied by adjusting the water–cement ratio and the Sp content. The viscosity of the cement paste was determined through the utilization of a rotational viscometer, while the fluidity of the cement paste was obtained through the utilization of the flow table test. When exploring the relationship between the viscosity of the slurry and particle movement, the fluidity of the slurry was controlled within the range of 300 ± 15 mm, while the viscosity of the cement paste was within the range of 0.2~1.2 Pa·s. In order to investigate the relationship between the flowability of the slurry and particle movement, the viscosity of the cement paste was then controlled within the range of 0.8 ± 0.1 Pa·s, while the fluidity of the cement paste was within the range of 160~320 mm, the vibration frequency was 200 Hz and the gravitational acceleration was set to 5G (average displacement—0.06 mm), and the vibration time was set to 2160 s. A relatively long period of time is used to increase the trend of particle movement. By controlling the fluidity, the relationship between viscosity and particle distribution trend can be obtained, the mixing ratios are listed in Table 1, then obtaining the relationship between the fluidity and the trend of particle distribution by controlling the viscosity, and the different mixing ratios are listed in Table 1.

In terms of the particles themselves, the particles are subjected to varying viscous forces during their movement in different cement paste environments. Therefore, at this stage the relationship between the rheology of the cement paste itself and the motion of the particles within the paste to obtain universal laws. Therefore the movement of particles in cement paste at different viscosities is studied, while the movement of particles in cement paste with different flowability was also tested.

**Figure 4:**
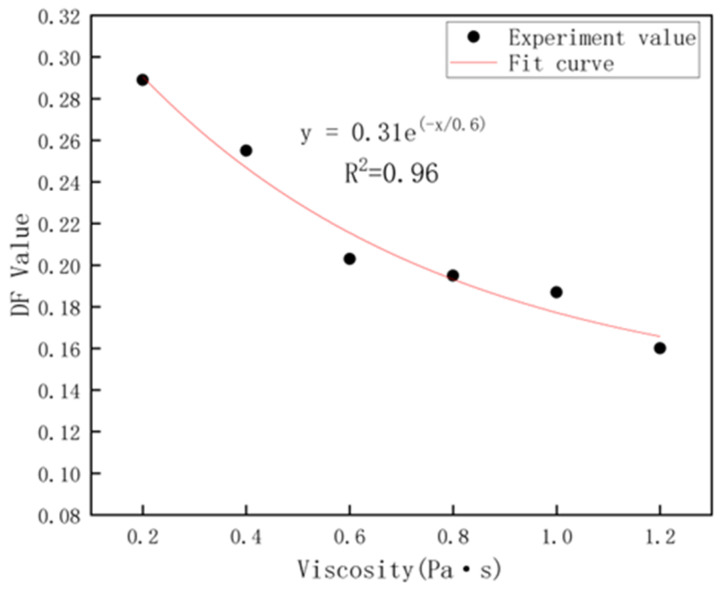
Relations between DF value and viscosity.

As can be seen from Figure 4, the degree of dispersion of the tagged particles presents a negative power function of the natural base with the viscosity. When the viscosity was 0.2 Pa·s, the value of the particle distribution dispersion degree (DF) was 0.29, indicating that there was a significant unevenness in the distribution of the tagged particles at this time, suggesting that the particles had undergone large-scale motion within the cement paste during this period, and as the viscosity increased, the DF value gradually decreased, indicating that particles became increasingly difficult to move in the higher viscosity environment. The ease of motion of the tagged particles is related to the viscosity of the cement paste. Previous research [11] has demonstrated that moving particles in non-coagulated cement paste experience friction. The strength of the adhesion between particles determines their ability to impede the flow of cement paste and results in a greater frictional force on particle movement. This adhesive property is known as viscosity. When subjected to vibration, the yield stress of cement paste decreases, making the motion of tagged particles easier. This motion is facilitated by a combination of factors, including the mixing action, gravity, frictional forces, and the viscosity of cement paste. The greater the frictional force on the tagged particles, the more challenging their motion becomes [25]. This is similar to the research conclusions drawn by previous scholars on the movement of aggregates and fibers, indicating that the primary mechanism of solid phase movement in cement-based slurries is similar [18,26,27].

**Figure 5:**
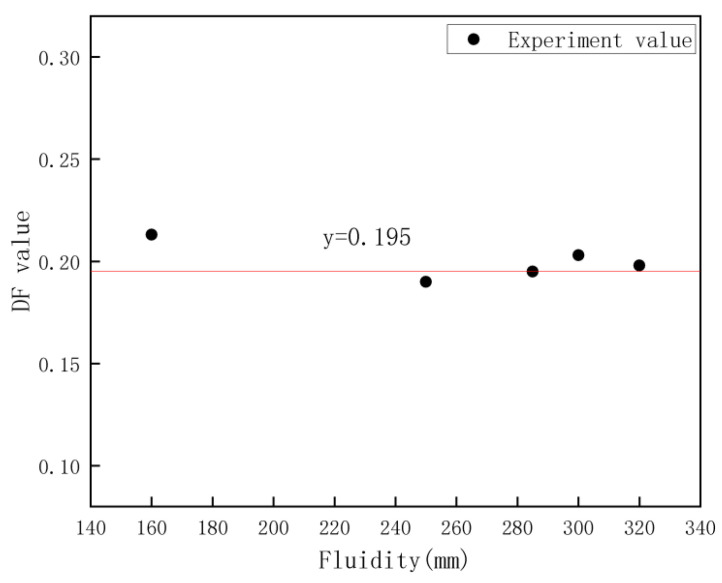
Relationship between DF value and the fluidity of cement paste after vibration.

The relationship between the DF value and the flowability of the cement paste can be seen in Figure 5, where all the data are around a horizontal straight line, which means that in the case of similar viscosity of the cement paste, even if there is a large difference in the flowability of the cement paste, the ease of movement of the marked particles in it is comparable in the vibrating state. The presence of a large number of hydrophilic groups on the surface of the composite particles and the strong ability of the hydrophilic groups to adsorb water molecules made the movement of the tagged particles in the cement paste easier as some of the particles in the cement paste became the bigger particles with a surface covered with water molecules. It leads to easier movement of the tagged particles in the cement paste in the low viscosity state, exhibiting a more uneven distribution of the tagged particles.

As a result, during the movement of the particles in the cement paste, the particles are obviously affected by the viscosity of the cement paste, especially when the viscosity is <1 Pa·s, the particles in the cement paste will move substantially when the cement paste is vibrated. Therefore, it is possible to control the viscosity of the cement paste to ensure that it can reduce the tendency of the cement paste to segregate even if it is subjected to different vibration conditions.

### 3.4. Significance of Particle Size and Density

The distribution trend of tagged particles with different densities and particle sizes in the cement slurry after vibration was studied in this section. Materials with different densities, which are characterized by their insolubility in water or non-reactivity with water, were used. Experiments were carried out in the same process as shown in Figure 1. Materials of different densities were divided into 75 μm ± 2 μm and 25 μm ± 2 μm through different sieves. The vibration frequency was 200 Hz, the gravitational acceleration was set to 5G (average displacement—0.06 mm), and the vibration time was set to 2160 s. The different materials ratios used in this stage are listed in Table 1. The proportion of tagged material in each group was controlled to 10% of the cement mass.

**Figure 6:**
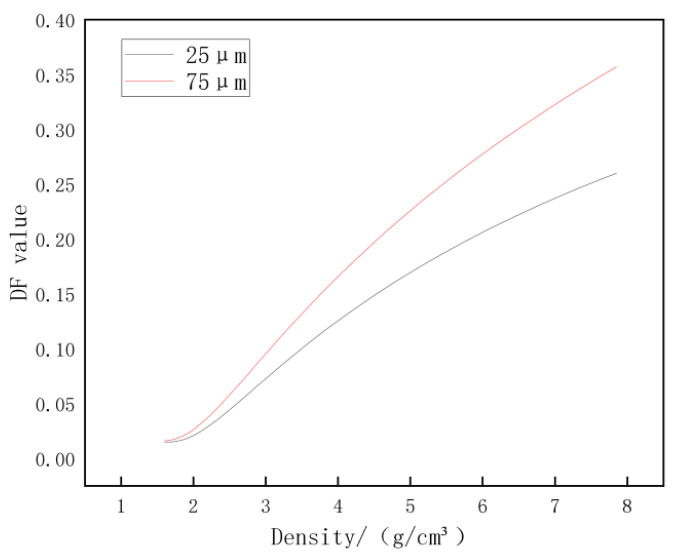
Effect of particle size and density on particle distribution under vibration.

Figure 6 shows the results of the distribution of tagged particles in the cement paste under vibration, as demonstrated by the different particle sizes and densities of the tagged material groups. It can be seen that in the case of using the same tagged material, the group with a large particle size showed a greater DF value compared to the group with a small size because the mass of the particles in the group with large-sized particles was greater compared to the group with small-sized particles. When the cement paste was vibrated, the raw material particles started to vibrate by the action of vibration. As the friction between the particles and the yield stress of the slurry started to drop, the tagged particles were subjected to the action of gravity and started to move. The particles of a larger size had stronger driving forces driving them to move at a higher speed. Therefore, a larger DF value is finally presented.

As for the density factor, for the groups with the same particle size, the denser material also had a higher mass, thus such a group also had a greater dispersion of tagged particles. By combining the relationship between dispersion and density at different particle sizes of the tagged materials and the movement of the tagged materials with different particle sizes, it was inferred that the dispersion of the tagged materials in the cement paste was closely related to their mass. In addition, it was observed that when the density of the tagged materials was less than 3 gr/cm³, a very small dispersion of the tagged particle distribution was observed. This was because, in low-density materials, the self-weight of the material and the effect of the driving force on the low-mass object could not overcome the effect of the frictional force due to the viscosity within the cement paste, resulting in a very low dispersion of the materials. It was widely recognized that cement-based materials with the addition of various fine powders had high strength and durability. From the perspective of the conclusion of this stage, the low-mass powder was tenaciously fixed in the cement paste and evenly distributed to fill the pores in the cement-based material, and this structure was difficult to be destroyed by external disturbances. As a result, this cement-based material had a more reliable compactness and exhibited great mechanical properties and durability. Furthermore, it can be seen from the results that even if the effect of the chemical reaction between the cementitious particles and water was not considered, there were still directional movements in the vibrating cement paste, which may have been related to the stronger water-secretion effect that occurred in the vibrated cement paste. Additionally, the solid–liquid mixture in the cement paste was separated due to the great density and mass differences between the gas phase and the solid–liquid mixture, so it was easy to understand that after vibration the cement-based material usually exhibited good particle buildup and better porosity [12].

In general, the method displayed can differentiate the distribution discreteness of particles with different densities and grain sizes in cement slurry, and the effect of these movements was related to the mass of the particles. The larger the mass, the stronger the movement dispersion effect would be. In addition, for these movements to occur, the particles needed to be larger than a certain density value of about 3 gr/cm³.

### 3.5. Effect of Vibration Frequency

In engineering practice, cement-based materials are subjected to vibrations over a wide frequency range, from several hertz to several hundred hertz [28,29]. Investigating the effects of different vibration frequencies on cement-based materials is crucial for understanding the underlying mechanisms of performance variations under dynamic conditions. Based on the above conclusions, the method mentioned above was used to evaluate the state of motion distribution of the particles at different frequencies. The different materials ratios are listed in Table 1. According to the conclusions of the above section, the viscosity of the different groups of cement slurries was regulated at the same level. The size of the particles of the tagged material used was controlled within the range of 25 ± 2 μm and 75 ± 2 μm by means of a sieve. Two size range groups were used to explore the differences between the different sizes of particles subjected to vibration in the cement paste. Different vibration frequencies are used to investigate the movement of particles in cement paste at different vibration frequencies. The frequencies were 1, 5, 35, 50, 100, 200, 500, 600, and 800 Hz. Vibration time was set to 2160 s.

**Figure 7:**
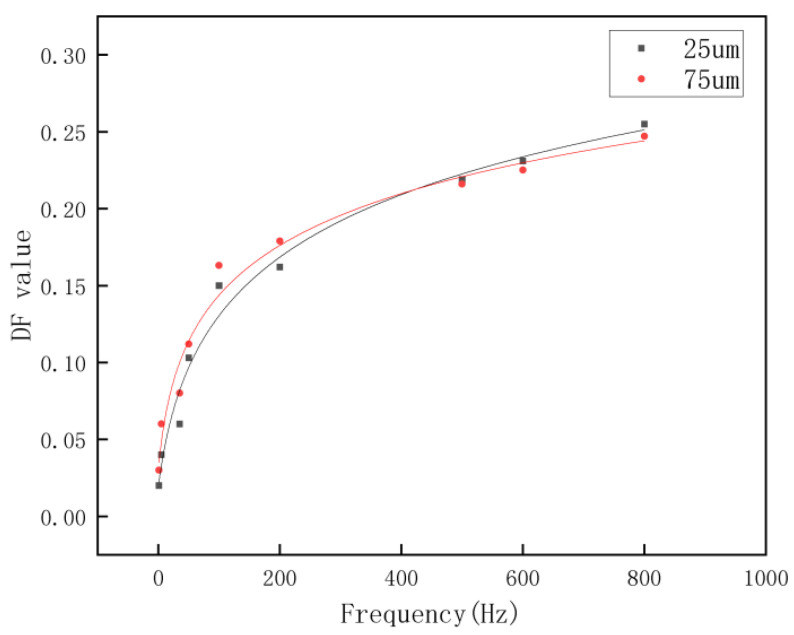
Effect of vibration frequency on particle distribution in vibration.

From Figure 7, it can be found that the DF values are slowly increasing at increasing vibration frequencies, which means that at higher frequencies, the particles in the cement paste produced more motion when subjected to vibration, leading to an increase in the inhomogeneity of the particle distribution, considering that vibration is an external influence factor on the cement paste and does not apply directly on the particles in the cement-based material, the process of vibration to which the tagged particles are subjected in the cement paste can be considered as a damped forced vibration model, while the externally applied vibration load is a simple harmonic load. In this stage, we investigate the effect of vibration frequency as a variable factor on the degree of particle dispersion in the cement-based material. For this process, the equation of motion for the damped forced vibration of the particles is in Equation (4):

(4) $$\ddot{y}=2\xi \omega \dot{y}+\omega^{2}y=\frac{F}{m}\sin\theta t,$$

*y* represents the displacement of the forced motion of the particle, *ξ* is the damping ratio of the cement paste, *ω* is the self-oscillation frequency of the particle, *Fsinθt* represents the magnitude of the simple harmonic force, and m represents the mass size of the object subjected to the forced vibration.

The solution of this equation is in Equations (5)–(7):

(5) $$y=\frac{F}{m\omega^{2}}\times \frac{1}{{\left(1-\frac{\theta^{2}}{\omega^{2}}\right)}^{2}+{\left(\frac{2\xi \theta }{\omega }\right)}^{2}}[(1-\frac{\theta^{2}}{\omega^{2}})\sin\theta t-\left(\frac{2\xi \theta }{\omega }\right)\cos\theta t],$$

Set:

(6) $$\;a=\sqrt{{\left(1-\frac{\theta^{2}}{\omega^{2}}\right)}^{2}+{\left(\frac{2\xi \theta }{\omega }\right)}^{2}},\;\tan\alpha =\frac{\frac{2\xi \theta }{\omega }}{\left(1-\frac{\theta^{2}}{\omega^{2}}\right)},\;\beta =\frac{1}{a},$$

Obtain:

(7) $$y=\frac{F}{m\omega^{2}}\times \beta \sin(\theta t-\alpha )$$

The observed increase in DF can be explained by the fact that below the resonant frequency, the dynamic coefficient tends to increase as the frequency increases. Furthermore, higher frequencies meant that in this vibration condition, the mixed phase followed to make higher frequency movements, the friction between particles drops lower, and the higher frequency vibration also meant that the motion of particles would be higher in frequency. That is to say that there will be more chance of movement in the cement paste, which explains why there is an elevated particle dispersion at higher frequencies. At vibration frequencies below 200 Hz, even though the distribution of the movement of larger particles and smaller particles in the cement paste after being subjected to vibration was relatively close, it can be seen that the dispersion of the larger-sized particles was still greater than that of the smaller particles, and with the increase in frequency, the dispersion of the smaller size tagged particles increased significantly and even surpassed that of the cement paste mixed with the larger-size tagged particles. Although the results were relatively similar for different-sized particle sizes in different groups, the repeated experiments showed the same pattern. This is that under the action of low frequency, the system vibrated very slowly, the displacement was synchronized with the load, the inertia and damping forces were small, and the dynamic load was mainly balanced by the elastic force (as illustrated in Equation (8)):

(8) $$\frac{F}{m\omega^{2}}=\frac{F}{K_{11}}\;K_{11}\;\text{is the stiffness coefficient of the material}$$

when the vibration frequency of the system was increasing, the stiffness coefficient of the system increased accordingly, the stiffness coefficient of the smaller-sized particles was smaller than that of the larger particle system, and the smaller size of the tagged material produced a larger deflection so that its degree of dispersion of distribution was increasing. It is predictable that the largest values of displacement excursions occur for both particles near the resonant frequency. The vibration frequency of the system keeps increasing, the dynamic coefficient of the system increases accordingly, the system starts to have a larger vibration, and the tagged materials produce a larger deflection so that the deflection coefficient DF keeps increasing. In a certain frequency from 500 to 800 Hz, the effect of the dynamic coefficient on the DF value is weaker than the effect of the stiffness coefficient on the DF value, and the lower particle size produces a larger displacement offset, making the lower particle size tagged materials exhibit a larger offset and show a larger DF value.

Based on the experimental results above, by using tagged particles, the motion patterns of cement particles under vibration were simulated. This method avoids the use of isotope labeling and large instruments, making it relatively cost-effective. The results can be quantified by weighing the particles compared to the visual tracer approach. Compared to using ping-pong balls, the tagged particles can be selected to match the size and density of cement particles, providing a more accurate reflection of the movement of cement particles in the slurry. By investigating the motion of microparticles in the vibrating cement slurry, this method allows us to explore the changes in the structure and mechanical properties of cement-based materials from the distribution of cement particles, which is more informative than investigating the distribution of fibers or aggregates. This is because the hydration products of cement particles are the most important part that supports the structure and strength of the entire cement-based material.

## 4. Conclusions

In this study, we developed a simulation method to analyze the movement of microparticles in vibrated cement paste, which is crucial for understanding the behavior of cement-based materials. This method provides a valuable tool for investigating the theory of particle motion in cement paste and complements previous research on the motion of microparticles in vibrated cement paste. Based on the results of the research process, the following conclusions can be obtained:

1. The proposed method shows the ideal feasibility of effectively simulating and quantifying the distribution of cement particles in cement slurry after vibration, which allows us to understand the distribution of the movement of the particles in cement slurry after vibration, and it is easy to implement, ensuring consistency between the collected material and the original added material.
2. The proposed method is able to quantify the discreteness in the distribution of particles between different densities and grain materials by the DF value, and it shows that during the vibration process, particles similar in size to cement particles maintain moving in a direction, but this trend gradually slows down. In order to ensure optimal handling of cement slurries subjected to prolonged vibration, it is necessary to carefully consider the selection of a slurry with appropriate viscosity.
3. The paste flowability alone does not contribute to the distribution pattern. The motion of cement particles of similar size in a vibrated cement slurry is limited by the viscosity of the slurry. The viscosity of the slurry and the dispersion degree of the tagged particles show a negative exponential change pattern. Maintaining high viscosity can well preserve the uniformity of the cement slurry.
4. The increase in frequency leads to a more pronounced degree of dispersion of the particles in the cement slurry. When utilizing vibration for processing cementitious materials with complex components, it is imperative to evaluate the impact of the vibration frequency on the homogeneity of the material.

## Figures and Tables

**Table 1:** Mix ratio of cement paste in this study.

Experiments	W/C	Dopant in Ratio to Cement				Size (μm)	Sp (%)
		SiC	Fe	B_4_C	C		
Feasibility Study	0.33	0.1	0.1	0.1	0.1	Original size	/
Effect of Vibration Time	0.3	0.1	/	/	/	Original size	0.8
Influence of Viscosity of Cement Paste	0.30	0.10	/	/	/	Original size	1
	0.25	0.11	/	/	/		2
	0.35	0.10	/	/	/		0.5
	0.30	0.12	/	/	/		0.8
	0.25	0.09	/	/	/		1.6
	0.25	0.09	/	/	/		1.2
Influence of Flowability of Cement Paste	0.25	0.09	/	/	/	Original size	0.9
	0.30	0.11	/	/	/		0.8
	0.35	0.12	/	/	/		0.6
	0.40	0.11	/	/	/		0.4
	0.50	0.09	/	/	/		0
Significance of Particle Size and Density	0.30	0.1				75	0.8
	0.33	0.1				25	0.9
	0.32		0.1			75	0.8
	0.33		0.1			25	0.8
	0.35			0.1		75	1
	0.36			0.1		25	0.8
	0.49				0.1	75	1
	0.52				0.1	25	1
Effect of Vibration Frequency	0.30	0.1	/	/	/	75	0.8
	0.33	0.1	/	/	/	25	0.9
